# Novel urinary protein biomarker panel for early diagnosis of gastric cancer

**DOI:** 10.1038/s41416-020-01063-5

**Published:** 2020-09-16

**Authors:** Takaya Shimura, Delphine Dayde, Hong Wang, Yusuke Okuda, Hiroyasu Iwasaki, Masahide Ebi, Mika Kitagawa, Tamaki Yamada, Tomonori Yamada, Samir M. Hanash, Ayumu Taguchi, Hiromi Kataoka

**Affiliations:** 1 Department of Gastroenterology and Metabolism, Nagoya City University Graduate School of Medical Sciences, 1 Kawasumi, Mizuho-cho, Mizuho-ku, Nagoya, 467-8601 Japan; 2 Department of Translational Molecular Pathology, The University of Texas MD Anderson Cancer Center, 1515 Holcombe Blvd, Houston, TX 77030 USA; 3 Department of Clinical Cancer Prevention, The University of Texas MD Anderson Cancer Center, 1515 Holcombe Blvd, Houston, TX 77030 USA; 4 Hangzhou Cosmos Wisdom Mass Spectrometry Center of Zhejiang University Medical School, 198 Qidi Road, Xiaoshan District, Hangzhou, Zhejiang China; 5 Department of Gastroenterology, Aichi Medical University, 1-1 Karimata, Iwasaku, Nagakute, 480-1195 Japan; 6 Okazaki Public Health Center, 1-3 Harusaki, Harisaki-cho, Okazaki, 444-0827 Japan; 7 Department of Gastroenterology, Japanese Red Cross Nagoya Daini Hospital, 2-9 Myoken-cho, Showa-ku, Nagoya, 466-0814 Japan; 8 Division of Molecular Diagnostics, Aichi Cancer Center, 1-1 Kanokoden, Chikusa-ku, Nagoya, Aichi 464-8681 Japan; 9 Division of Advanced Cancer Diagnostics, Nagoya University Graduate School of Medicine, 65 Tsurumai-cho, Showa-ku, Nagoya, Aichi 466-8550 Japan

**Keywords:** Gastric cancer, Diagnostic markers

## Abstract

**Background:**

With the goal of discovering non-invasive biomarkers for early diagnosis of GC, we conducted a case-control study utilising urine samples from individuals with predominantly early GC vs. healthy control (HC).

**Methods:**

Among urine samples from 372 patients, age- and sex-matched 282 patients were randomly divided into three groups: 18 patients in a discovery cohort; 176 patients in a training cohort and 88 patients in a validation cohort.

**Results:**

Among urinary proteins identified in the comprehensive quantitative proteomics analysis, urinary levels of TFF1 (uTFF1) and ADAM12 (uADAM12) were significantly independent diagnostic biomarkers for GC, in addition to *Helicobacter pylori* status. A urinary biomarker panel combining uTFF1, uADAM12 and *H. pylori* significantly distinguished between HC and GC patients in both training and validation cohorts. On the analysis for sex-specific biomarkers, this combination panel demonstrated a good AUC of 0.858 for male GC, whereas another combination panel of uTFF1, uBARD1 and *H. pylori* also provided a good AUC of 0.893 for female GC. Notably, each panel could distinguish even stage I GC patients from HC patients (AUC = 0.850 for males; AUC = 0.845 for females).

**Conclusions:**

Novel urinary protein biomarker panels represent promising non-invasive biomarkers for GC, including early-stage disease.

## Background

Gastric cancer (GC) is the fifth most common malignancy and the third leading cause of cancer deaths worldwide,^1^ and this high mortality has continued despite a recent plateau in incidence. The 5-year survival rate for GC is greater than 90% for stage I, in contrast to 15% for stage IV with distant metastasis.^2^ Early diagnosis is clearly critically important to achieve curability for GC. Endoscopy with pathological diagnosis from a biopsy sample is currently the gold standard for diagnosing GC but is unsuitable for mass-screening due to the high invasiveness, risk, and financial and time costs. Elucidation of non-invasive diagnostic biomarkers for GC is thus needed to improve prognosis.

*Helicobacter pylori* (*H. pylori*) antibody has been used as a serological test for GC screening, because atrophic gastritis through *H. pylori* infection is one of the major causes of GC.^3^ Testing for *H. pylori* antibody might be useful to identify the high-risk group with atrophic gastritis, but screening for active infection of *H. pylori* is insufficient for diagnosing GC because of the high false-positive rate and *H. pylori* cannot remain in the stomach with severe atrophic gastritis. Testing with *H. pylori* antibody is thus not recommended for population-based screening due to insufficient evidence. It may offer a good predictor of future GC development, but does not provide a suitable indicator of current GC.

Serum tumour markers, such as carcinoembryonic antigen (CEA) and carbohydrate antigen 19-9 (CA19-9), have often been used in clinical practice, but their utilisation has not been recommended for diagnosis of GC because of the low sensitivity, at <20% for early-stage disease and 20–50% for advanced stage.^4^ Moreover, concentrations of these tumour markers never increase in early-stage GC, such as stage I. Hence, clinically applicable biomarkers for early detection of GC have been lacking and the discovery of non-invasive biomarkers is anticipated to facilitate a high curability for GC.

As a completely non-invasive sample, urine sample is very suitable for mass-screening test. However, no urinary biomarkers have been utilised to the clinical diagnosis of malignancies. We have previously reported the usefulness of urinary biomarkers for diagnosing GC and colorectal cancer (CRC)^5–9^ and have established methods for handling urine samples. We demonstrate herein the utility of a urinary protein biomarker panel for detecting GC.

## Methods

### Patients and study design

All samples were collected between September 2012 and June 2017 from threee participating Japanese institutions. Patients between 20 and 90 years old were recruited for this study. The inclusion criteria for the GC group were adenocarcinoma histologically confirmed from endoscopic biopsy and no treatment before study enrolment. The inclusion criteria for the healthy control (HC) group were no evidence of neoplasms at their annual check-up. Patients with a history of neoplasm of any type and/or with multiple neoplasms were excluded from enrolment in this study.

To ensure the accuracy and comprehensiveness of reporting in this case-control biomarker study, the present study complied with both the REMARK guidelines^10^ and the STROBE statement.^11^ The study protocol was approved by the ethics committee at each participating institution and was conducted according to the ethical guidelines of the Helsinki Declaration of 1964 and later versions. All patients provided written, informed consent prior to enrolment in the study. This study was registered to the University Hospital Medical Information Network Clinical Trials Registry (UMIN000021350).

### Samples and definition

All urine and serum samples were collected before any treatment for GC, divided into small aliquots, immediately frozen and stored at −80 °C until assayed, as previously reported.^8,9^ Urine samples were thawed on ice and centrifuged to remove precipitates before analysis. Clinical stage was determined by the final pathological diagnosis after resection, according to the 7th edition of the Union for International Cancer Control tumour-node-metastasis classification.^12^ *H. pylori* status was analysed using the serum or urinary anti-*H. pylori* immunoglobulin (Ig)G antibody, and successful eradication for *H. pylori* was analysed by the ^13^C-urea breath test. Patients who were formerly positive for *H. pylori* but whose infection was successfully eradicated were also included in a negative group of *H. pylori*.

### Mass spectrometry analysis

Protein concentrations in urine samples were determined by Bradford assay (Coomassie Protein Assay Reagent; ThermoFisher Scientific, Waltham, MA). Proteins in each pooled urine samples were reduced with 20 mM tris(2-carboxyethyl)phosphine (TCEP), followed by alkylation with 50 mM acrylamide and in-solution tryptic digestion. The mixture of labelled samples was separated into 24 fractions using high-pH reversed-phase chromatography (RPLC) on a high-performance liquid chromatography (HPLC) system (Shimadzu) with a reversed-phase column (RPGS, 4.6 × 150 mm, 15 µm, 300 Å) and lyophilised. The dried fractions were resolved in 0.1% TFA for nano Liquid Chromatography Mass Spectrometry (LC-MS) analysis.

Gradient elution was performed for each of the 24 fractions in a capillary column (75 μmID × 300 mm length, C18 with 3 µm particle size and 100 A pore size; Column Technology, Chicago, IL) at 500 nl/min with mobile phases A (0.1% formic acid (FA) in 2% acetonitrile (ACN)) and B (0.1% FA in 98% can). Quantitative MS analysis for each protein fraction was performed in the QExactive Mass Spectrometer (ThermoFisher Scientific) in data-dependent acquisition (DDA) mode. Survey scan MS was acquired in 400–1800 *m/z* range with resolution of 70,000 at *m/z* 400 and an automatic gain control (AGC) target value of 1 × 10^6^ ions with a 100-ms maximum injection time. The most 10 intense ions (>5000 counts) with charge states +2 to +4 were sequentially isolated and fragmented in the high collision energy (HCD) cell with stepped collision energy (20, 25, and 27 normalised collisional energy (NCE)) and a maximum injection time of 150 ms at a target value of 5 × 10^5^ ions with resolution of 17,500. Polydimethylcyclosiloxane ions generated in the electrospray process from ambient air (protonated (Si(CH_3_)_2_O))_6_; *m/z* = 445.120025) were used as the lock-mass for internal recalibration in real time.

Acquired spectra were searched against the complete proteome set of *Homo sapiens* from UniProt (released version 2017_01). Database search parameters were set as follows: a maximum of two missed cleavage sites permitted for trypsin digestion, 10-ppm precursor mass tolerance, 0.05-Da fragment mass tolerance, propionamide modification for cysteine (+71.037 Da), TMT 6-plex modification for N-terminal (+229.163 Da) and lysine (+229.163 Da) as the fixed modifications, and oxidation modification for methionine (+15.995 Da) as a dynamic modification. LC-MS/MS data were analysed using Protein Discovery version 1.4 software (ThermoFisher Scientific). All searches were filtered to a <1% false discovery rate (FDR), and the relative quantification result was normalised to protein level.

### Enzyme-linked immunosorbent assays (ELISAs)

We measured urinary protein concentrations for each of the proteins of interest using monospecific ELISAs, in accordance with the instructions from the manufacturers. To measure each urinary protein concentration, we used the respective ELISA kits for trefoil factor 1 (TFF1) (#EK1232; Boster Biological Technology, Pleasanton, CA), a disintegrin and metalloproteinase domain-containing protein 12 (ADAM12) (#.DAD120; R&D Systems, Minneapolis, MN), pepsinogen 3 (PGA3) (#OKEH03169; AVIVA SYSTEMS BIOLOGY, San Diego, CA), BRCA1-associated RING domain 1 (BARD1) (#.MBS7236583; MyBioSource, San Diego, CA), coiled coil domain-containing protein 38 (CCDC38) (#.MBS7209768; MyBioSource), tubulointerstitial nephritis antigen-like 1 (TINAGL1) (#MBS9340283; MyBioSource), DEAD-box helicase 55 (DDX55) (#MBS7244207; MyBioSource) and a Parameter Creatinine Assay (R&D Systems) for creatinine. Each ELISA analysis required 1–100 μl of urine or serum sample.

### Statistical analysis

The primary endpoint of the present study is to establish urinary protein biomarker panel for detecting the presence of GC. Representative variables were described with mean or median values and analysed using the *t*-test or Mann–Whitney U test, as appropriate. Other data were analysed using the Chi-squared test or Fisher’s exact probability test, as appropriate. The nonparametric Spearman’s rank correlation coefficient (r) was used as a measure of correlation. We randomly matched between two groups, using the propensity score including two factors (age and sex). Receiver operating characteristic (ROC) curve analysis was used to calculate the area under the curve (AUC) with 95% confidence interval (CI) for each biomarker. According to AUC values, diagnostic accuracy was classified into five grades: 0.90–1.0, excellent; 0.80–0.90, good; 0.70–0.80, fair; 0.60–0.70, poor; 0.50–0.60, fail. Logistic regression modelling was used to estimate the odds ratio (OR) with 95%CI and to construct a composite score, which was used to calculate the AUC for combination biomarkers. Each cut-off value was determined from Youden’s index on the ROC curves. All statistics were calculated using SPSS Statistics version 25 (IBM Corp., Tokyo, Japan). A two-tailed *P* < 0.05 was considered statistically significant.

## Results

### Patients

**Fig. 1:**
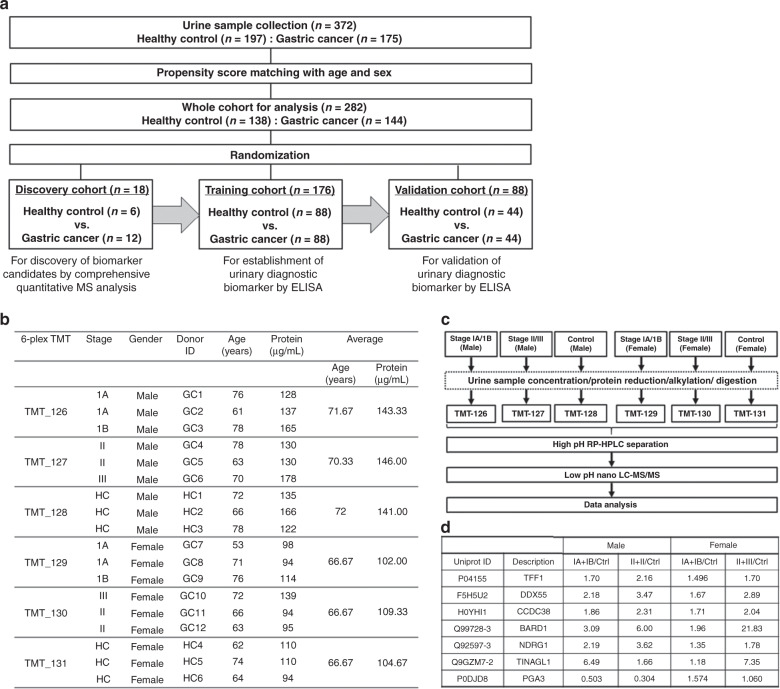
Study flowchart and quantitative mass spectrometry. **a** Consort diagram; **b** Information on individual donor, sample pooling and TMT label; **c** High-throughput quantitative proteomics platform for cancer biomarker discovery; **d** Urinary protein with aberrant expression in gastric cancer patients. MS mass spectrometry, ELISA enzyme-linked immunosorbent assay, TMT tandem mass tag, GC gastric cancer, HC healthy control, IA + IB/Ctrl ratio of urinary protein in GC patients with stage IA or IB to that in HCs, II + III/Ctrl ratio of urinary protein in GC patients with stage II or III to that in HCs.

In total, 372 patients were enrolled from September 2012 to June 2017 at three Japanese institutions, comprising 197 patients with HC and 175 patients with GC. After PS matching, 282 patients were selected as a whole cohort in the present study. The whole cohort was randomly divided into three groups: 18 patients in the discovery cohort; 88 pairs (176 patients) in the training cohort and 44 pairs (88 patients) in the validation cohort. Urinary biomarkers were identified in the discovery cohort, a biomarker panel was established in the training cohort, then established biomarkers were tested in the independent validation cohort (Fig. 1a).

### Identification of urinary protein biomarkers by quantitative proteomics analysis

To discover urinary diagnostic biomarkers for GC, we have developed a high-throughput quantitative proteomics platform employing the 6-plex TMT labelling approach, together with orthogonal two-dimensional RPLC-MS/MS analysis for cancer biomarker discovery. We undertook an in-depth proteomics analysis of urine specimens derived from patients with GC and matched HCs. Six pooled urine samples with an average concentration of 124.4 µg/ml were prepared from the three individuals in each subgroup (Fig. 1b). Samples were then labelled with 6-plex tandem mass tags (TMT) reagent and combined (Fig. 1c). Data obtained from LC-MS/MS analysis were processed to calculate the fold changes as a normalised ratio for cancer compared to healthy subjects. This study resulted in the quantification of 1148 proteins encoded by 1112 unique genes. Among urinary proteins with aberrant expression in the comprehensive proteomics analysis, we finally selected seven urinary protein candidates (TFF1, PGA3, BARD1, CCDC38, TINAGL1, NDRG1 and DDX55) for next-step analysis, which fulfilled the following criteria from previous reports and databases including The Cancer Genome Atlas, The Human Protein Atlas and GeneCards: protein with small molecular weight; and cancer/testis antigen or proteins specifically expressed in stomach (Fig. 1d). We also analysed urinary levels of ADAM12, which was identified as a good urinary biomarker for GC in our small sample pilot study.^9^

### Establishment of urinary biomarkers

As shown in Table 1, baseline characteristics were well balanced between both HC and GC groups. No significant differences in age, sex or serum creatinine were found between groups, in either training or validation cohorts. In the HC group, 16 patients were *H. pylori* positive (12 for serum test and 4 for urinary test), 47 patients were *H. pylori* negative with eradication (breath test) and 69 patients were *H. pylori* negative never infected (36 for serum test and 33 for urinary test). In the GC group, 64 patients were *H. pylori* positive (32 for serum test and 32 for urinary test), 14 patients were *H. pylori* negative with eradication (breath test), 50 patients were *H. pylori* negative never infected (22 for serum test and 28 for urinary test) and 4 patients were unknown *H. pylori* status. Expectedly, positivity for *H. pylori* was higher in the GC group than in the healthy group (*P* < 0.001). The training and validation cohorts showed even distributions of every stage.

**Table 1:** Background characteristics of the study cohort.

	Training cohort (*n* = 176)		*P*	Validation cohort (*n* = 88)		*P*
	HC	GC		HC	GC	
	(*n* = 88)	(*n* = 88)		(*n* = 44)	(*n* = 44)	
Age (years)						
Median (range)	68 (38–83)	69 (42–85)	0.550^a^	68 (40–88)	68 (45–77)	0.960^a^
Sex						
Male	67	63	0.493^b^	34	36	0.597^b^
Female	21	25		10	8	
Serum Cr (mg/dl)						
Mean ± SD	0.82 ± 0.17	0.81 ± 0.22	0.669^b^	0.79 ± 0.13	0.86 ± 0.30	0.154^c^
*H. pylori*						
Positive	11	39	<0.001^b^	5	25	<0.001^b^
Negative	77	(46)		(39)	(18)	
(Eradicated)	(−30)	(−12)		(−17)	(−2)	
(Never infected)	(−47)	(−34)		(−22)	(−16)	
Unknown	0	3		0	1	
Histology						
Intestinal type		57 (64.8%)			31 (70.5%)	
Diffuse type		31 (35.2%)			13 (29.5%)	
Stage, *n*						
I		54 (61.4%)			30 (68.2%)	
II		10 (11.4%)			3 (3.4%)	
III		8 (9.1%)			4 (9.1%)	
IV		16 (18.2%)			7 (15.9%)	

*H. pylori*-negative includes individuals who have undergone successful eradication. Clinical stage is according to the 7th edition of the UICC-TNM classification.

*GC* gastric cancer, *HC* healthy control, *n* number, *Cr* creatinine.

^a^Mann–Whitney U test.

^b^χ^2^ test.

^c^*t*-test.

Urinary concentrations of eight candidate proteins were analysed by quantitative monospecific ELISAs in the training cohort. All urinary protein levels were normalised to the urinary total protein level. Among these eight proteins, urinary levels of TFF1 (uTFF1), ADAM12 (uADAM12), PGA3 (uPGA3) and BARD1 (uBARD1) were significantly higher in the GC group than in the HC group on univariate analysis. Multivariate analysis identified uTFF1 and uADAM12 as independent significant proteins for the diagnosis of GC (uTFF1: OR 1.033, 95% CI, 1.011–1.055, *P* = 0.003; uADAM12: OR 1.026, 95% CI 1.009–1.043, *P* = 0.003) as well as *H. pylori*-positive status (OR 3.717, 95% CI 1.502–9.259, *P* = 0.005), but uPGA3 and BARD1 were not significant. As a result, uTFF1, uADAM12 and *H. pylori*-positive status were considered as provisional diagnostic biomarkers for GC (Table 2).

**Table 2:** Urinary protein level.

Training cohort (*n* = 176)						
		Univariate analysis			Multivariate analysis	
		HC (*n* = 88) (Median (IQR))	GC (*n* = 88) (Median (IQR))	*P*	Odds ratio (95% CI)	*P*
TFF1	(pg/μg)	5.4 (3.2–13.8)	30.7 (10.1–74.5)	<0.001	1.033 (1.011–1.055)	0.003
ADAM12	(pg/μg)	19.5 (10.3–30.1)	37.4 (20.0–61.6)	<0.001	1.026 (1.009–1.043)	0.003
PGA3	(pg/μg)	3.4 (1.0–6.3)	5.7 (2.3–27.5)	0.002		
BARD1	(pg/μg)	28.3 (17.4–49.6)	41.5 (27.5–72.2)	0.003		
CCDC38	(pg/μg)	36.1 (20.2–52.3)	37.5 (25.6–73.7)	0.162		
TINAGL1	(pg/μg)	0.0 (0.0–1.3)	0.0 (0.0–2.2)	0.873		
NDRG1	(pg/μg)	225 (122–504)	325 (156–465)	0.576		
DDX55	(pg/μg)	57.1 (31.8–83.8)	59.3 (37.1–133.1)	0.089		
*H. pylori*				<0.001	3.717 (1.502–9.259)	0.005

Validation cohort (*n* = 88)						
		Univariate analysis			Multivariate analysis	
		HC (*n* = 44) (Median (IQR))	GC (*n* = 44) (Median (IQR))	*P*	Odds ratio (95% CI)	*P*
TFF1	(pg/μg)	9.2 (5.3–18.0)	54.9 (21.3–93.9)	<0.001	1.010 (0.997–1.023)	0.136
ADAM12	(pg/μg)	19.7 (12.7–33.5)	32.5 (22.2–54.8)	0.002	1.034 (1.006–1.064)	0.018
*H. pylori*				<0.001	11.764 (3.472–40.000)	<0.001

Each urinary level was normalised with urinary protein concentration.

**Fig. 2:**
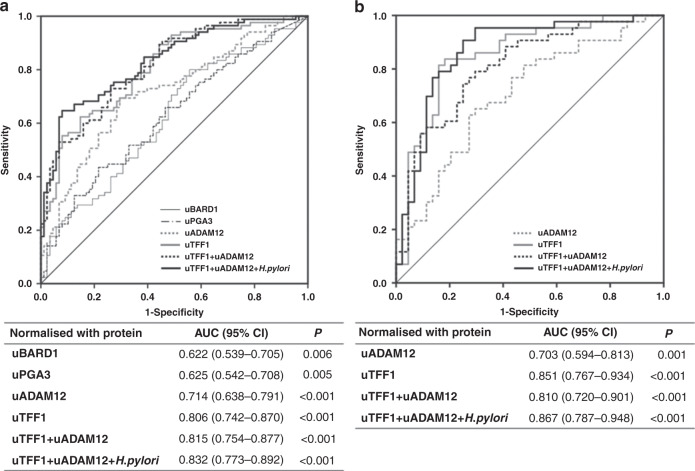
Receiver operating characteristic (ROC) curves. **a** Training cohort, **b** Validation cohort. ROC curves were obtained from values normalised to urinary total protein.

Moreover, uTFF1, uADAM12, uPGA3 and uBARD1 allowed significant differentiation between HC and GC groups in ROC analyses for the training set (uTFF1: AUC = 0.806; uADAM12: AUC = 0.714; uPGA3: AUC = 0.625; uBARD1: AUC = 0.622). When uTFF1 was combined with uADAM12 and *H. pylori*, these combination biomarkers distinguished between HC and GC (uTFF1 + uADAM12: AUC = 0.815, 95% CI 0.754–0.877; uTFF1 + uADAM12 + *H.pylori*: AUC = 0.832, 95% CI, 0.773–0.892) (Fig. 2a). These results suggested that a urinary biomarker panel combining uTFF1, uADAM12 and *H. pylori* status might provide a good diagnostic biomarker for GC. When urinary protein levels were normalised to urinary creatinine, similar results were obtained: uTFF1, uADAM12 and uPGA3 were significant biomarkers on univariate analysis and uTFF1, uADAM12 and *H. pylori*-positive status keeping as independent biomarkers on multivariate analysis, had an AUC of 0.777 (95% CI 0.709–0.845) in a combining panel for the training cohort (Supplementary Table S1, Supplementary Fig. S1a).

### Validation of urinary biomarkers

Next, we examined the diagnostic quality of established biomarker panels in the independent validation cohort. As shown in Table 2, uTFF1 and uADAM12 were significantly higher in the GC group than in the HC group, in the validation cohort. ROC analysis of a urinary biomarker panel combining uTFF1 and uADAM12 for the validation cohort also showed significant differentiation between HC and GC groups (uTFF1: AUC = 0.851, 95% CI 0.767–0.934) (uADAM12: AUC = 0.703, 95% CI 0.594–0.813). A biomarker panel combining uTFF1, uADAM12 and *H. pylori* revealed a good AUC of 0.867 (95% CI 0.787–0.948), as good as in the training cohort (Fig. 2b). An AUC of this combination biomarker panel was much higher than that of *H. pylori* status alone (AUC = 0.689, 95% CI 0.624–0.755). When urinary protein levels were normalised to urinary creatinine, uTFF1 and uADAM12 were the independent diagnostic biomarkers for GC (Supplementary Table S1), and a biomarker panel combining uTFF1, uADAM12 and *H. pylori* revealed also a good AUC of 0.863 (95% CI 0.787–0.939) (Supplementary Fig. S1b).

Moreover, uTFF1 and uADAM12 showed significantly higher levels in the GC group than in the HC group, regardless of GC histological type (Supplementary Fig. S2a, b). Additionally, we analysed another subset analysis, according to *H. pylori* status dividing the whole cohort into *H. pylori*-positive and -negative groups. In the *H. pylori*-positive group, both uTFF1 and uADAM12 were significantly higher in the GC group than in the HC group (median uTFF1: 22.1 pg/μg protein for HC *vs*. 44.6 pg/μg protein for GC; *P* = 0.034) (median uADAM12: 21.2 pg/μg protein for HC vs. 27.9 pg/μg protein for GC; *P* = 0.036). In the *H. pylori*-negative group, both uTFF1 and uADAM12 were also significantly higher in the GC group than in the HC group (median uTFF1: 6.5 pg/μg protein for HC vs. 25.6 pg/μg protein for GC; *P* < 0.001) (median uADAM12: 19.3 pg/μg protein for HC vs. 40.4 pg/μg protein for GC; *P* = 0.036).

Even if *H. pylori* status was subcategorised into three groups: *H. pylori* positive; *H. pylori* eradicated; *H. pylori* never infected, both uTFF1 and uADAM12 were significantly independent from *H. pylori* status (Supplementary Table S2). A biomarker panel combining uTFF1, uADAM12 and the three subcategorised *H. pylori* status also revealed a good AUC of 0.840 (95% CI 0.791–0.889) in the subset analysis according to *H. pylori* subcategorisation (Supplementary Fig. S3a).

### Sex-specific biomarkers

**Fig. 3:**
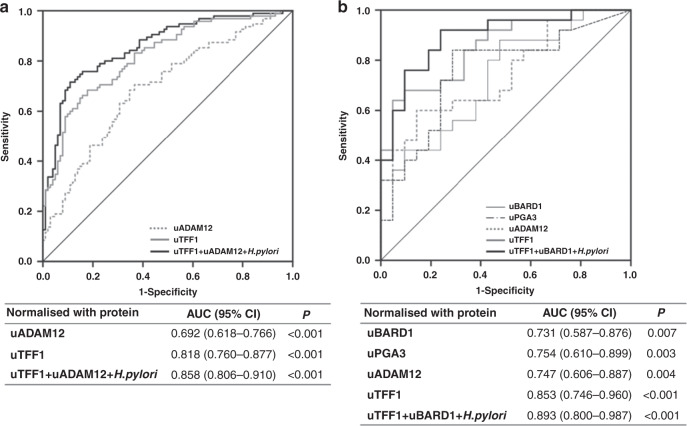
Sex-specific urinary biomarker panels. **a** Male cohort, **b** Female cohort. Subset analysis was performed according to sex. ROC curves were obtained from values normalised to urinary total protein.

To identify sex-specific urinary biomarkers, we performed subset analysis according to sex. Dividing the whole cohort into male and female cohorts, uTFF1, uADAM12 and *H. pylori* offered significantly independent biomarkers for male GC, whereas uTFF1, BARD1 and *H. pylori* were significantly independent biomarkers for female GC (Supplementary Table S3). A urinary biomarker panel combining uTFF1, uADAM12 and *H. pylori* significantly distinguished between HC and GC groups in the male cohort, with a good AUC of 0.858 (95% CI 0.806–0.910) (Fig. 3a). As for the female cohort, a urinary biomarker panel comprising uTFF1, BARD1 and *H. pylori* also showed a good AUC of 0.893 (95% CI 0.800–0.987) (Fig. 3b). This sex-specific combination urinary biomarker panel also showed 86.7% sensitivity, 61.4% specificity and 73.8% accuracy (Supplementary Table S4), which showed much higher sensitivity than conventional serum tumour markers, CEA (15.0%) and CA19-9 (16.8%).

Next, we also analysed serum levels of TFF1 (sTFF1), ADAM12 (sADAM12) and BARD1 (sBARD1), using 93 serum samples paired to urine samples in this study. sTFF1 and sBARD1 showed significantly higher levels in the GC group than in the HC group, but sADAM12 did not (Supplementary Table S5). No significant correlations were found between serum and urinary levels. AUCs of sTFF1, sADAM12 and sBARD1 were 0.676, 0.410 and 0.703, respectively. Urinary levels of these three proteins showed better diagnostic performance than serum levels of those.

### Early discovery

**Fig. 4:**
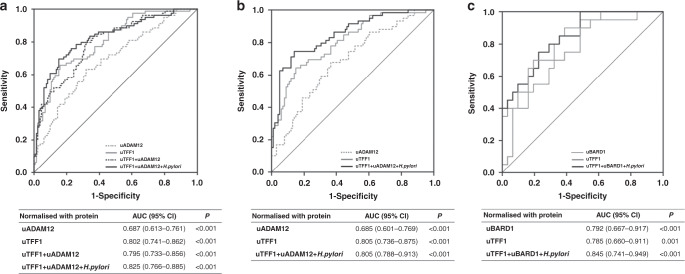
Early detection of stage I GC. **a** Whole cohort, **b** Male cohort, **c** Female cohort. ROC curves were obtained from values normalised to urinary total protein to distinguish stage I GC patients from healthy controls.

Comparing HC with stage I GC, both uTFF1 and uADAM12 were significantly higher in the stage I GC group than in the HC group (uTFF1, *P* < 0.001; uADAM12, *P* < 0.001). A biomarker panel combining uTFF1, uADAM12 and *H. pylori* even distinguished between HC and stage I GC, with a good AUC of 0.825 (95% CI 0.766–0.885) (Fig. 4a). The significance of this combination biomarker panel was not changed in the subset analysis after subcategorisation into *H. pylori* positive, *H. pylori* eradicated and *H. pylori* never infected (Supplementary Fig. S3b). Moreover, urinary biomarker panels for male GC (uTFF1/uADAM12/*H. pylori*) and female GC (uTFF1/uBARD1/*H. pylori*) could diagnose stage I GC with good AUCs (male GC panel: uTFF1/uADAM12/*H. pylori* = 0.850, 95% CI 0.788–0.913; female GC panel: uTFF1/uBARD1/*H. pylori* = 0.845, 95% CI 0.741–0.949) (Fig. 4b, c). Surprisingly, these urinary biomarker panels showed potential diagnostic value even for stage I GC, with 91.5% sensitivity and 52.5% specificity for males and 100% sensitivity and 51.6% specificity for females, which showed much higher sensitivity than conventional serum tumour marker CEA (10.1%) and CA19-9 (4.6%) in this study.

### Disease stage

Finally, we analysed uTFF1, uADAM12 and uBARD1 according to disease stage. As shown in Supplementary Fig. S4a, b, no significant correlations were found between disease stage and uTFF1/uADAM12 (uTFF1: r = 0.106, *P* = 0.224; uADAM12: r = 0.132, *P* = 0.150). Median levels of uTFF1 were 7.5 pg/μg protein for HC, 29.3 pg/μg protein for stage I GC, 36.1 pg/μg protein for stage II/III GC and 50.0 pg/μg protein for stage IV GC. Median levels of uADAM12 were 19.5 pg/μg protein for HC, 30.5 pg/μg protein for stage I GC, 34.0 pg/μg protein for stage II/III GC and 51.4 pg/μg protein for stage IV GC. uTFF1 and uADAM12 were significantly higher in all stage GC groups than in the HC group (*P* < 0.001).

On the other hand, uBARD1 displayed significant negative correlations with disease stage (uBARD1: r = −0.519, *P* = 0.002) (Supplementary Fig. S4c). Median levels of uBARD1 were 23.8 pg/μg protein for HC, 52.7 pg/μg protein for stage I GC, 41.5 pg/μg protein for stage II/III GC and 15.7 pg/μg protein for stage IV GC. The stage I GC group showed significantly higher level of uBARD1 compared to the HC (*P* < 0.001) and stage IV GC (*P* = 0.001) groups.

## Discussion

This study established a novel non-invasive potential diagnostic biomarker panel for GC, comprising uTFF1, uADAM12 and *H. pylori* status for males and uTFF1, uBARD1 and *H. pylori* status for females. Of note, the present biomarker panel could distinguish even early-stage GC with high accuracy. Urine sampling is a non-invasive procedure with no risk and low cost, representing an attractive option for biomarker detection. In fact, we have previously shown the utility of urinary biomarkers including protein and miRNA for detecting GC^7,9^ and colorectal cancer,^6^ as well as curability of GC.^8^ We identified novel urinary biomarker panels offering good diagnostic power, using a straightforward method comprising biomarker discovery, establishment and validation in the large cohort.

TFF1 was the most robust diagnostic biomarker for GC in this study. TFF1 is a member of the trefoil factor family, comprising three members of TFF1, TFF2 and TFF3. These small, secreted molecules are mainly expressed in gastrointestinal epithelial cells and play roles in mucosal repair and mucus polymerisation.^13^ Among the TFF family, TFF1 is mainly produced in superficial cells of the stomach body and antral mucosa.^13^ TFF1 is considered a gastric-specific tumour suppressor gene^14^ and immunoreactivity to TTF1 was detected more often in GC tissues than in normal tissues in previous studies.^15,16^ Recent studies have shown that loss of TFF1 induces gastric carcinogenesis through activation of proinflammatory signals including nuclear factor-kappa B (NF-κB)^17^ and signal transducer and activator of transcription 3 (STAT3).^18^ Serum level of TFF1 was a diagnostic biomarker for GC patients and *H. pylori* infection.^19^ Another study also showed that TFF1 was useful for detecting disseminated GC cells in peripheral blood and bone marrow.^20^ Indeed, urinary level of TFF1 showed higher tendency in *H. pylori*-positive HCs compared to *H. pylori*-negative HCs, consistent with previous results. However, in both *H. pylori*-positive and -negative status, urinary levels of TFF1 were significantly higher in the GC patients than in HCs. In addition, uTFF1 was independent from currently and previously *H. pylori* infection on multivariate analysis. Hence, uTFF1 offered the most powerful diagnostic biomarker for GC in the current study. Detection of tiny changes in urinary protein level would presumably result in more delicate sensitivity of this biomarker. Although a few reports have examined uTFF1 as a biomarker of chronic kidney disease,^21,22^ no studies have been reported for urinary biomarkers of GC. Urinary assay of TFF1 is thus promising for detecting GC according to the results of our study.

In terms of ADAM12, a family of metalloprotease-related metalloproteinases, we have demonstrated that ADAM12-mediated HB-EGF (heparin binding-epidermal growth factor-like growth factor) shedding played a critical role in GC development in a preclinical model.^23,24^ In addition, we also previously reported that ADAM12 was highly expressed in GC tissues and offered a diagnostic biomarker for GC in another pilot study with a small sample size (*N* = 70).^9^ The AUC of uADAM12 was 0.757 in that pilot study, similar to the present data from our large-cohort study. Moreover, the present study found that uADAM12 could contribute to the detection of early-stage GC, although its significance was independent only for the male cohort. Since males are well known to be predominant in the GC population, the significance of uADAM12 in male GC patients might contribute its significance in whole cohort analysis.

In contrast, uBARD1 was an independent diagnostic biomarker for female GC in the present study. BARD1 interacts with BRCA1, in which germline mutation causes familial breast and ovarian cancers, and these form a heterodimer complex that plays a role as a tumour suppressor.^25^ BARD1 also induces apoptosis through p53 accumulation independent of BRCA.^26^ On the other hand, protein and RNA of BARD1 were overexpressed in the cytoplasm of ovarian, breast and lung cancer tissues compared to normal tissues.^27^ Similarly, overexpression of BARD1 was observed in tissues from hepatocellular carcinoma^28^ and colorectal cancer.^29^ From these results, BARD1 is also considered to play an oncogenic function. However, no previous reports have described the relationship between BARD1 and GC. Moreover, no studies have investigated circulating levels of BARD1, including in serum and urine, although one study analysed serum autoantibody against BARD1 as a biomarker of lung cancer,^30^ not BARD1 itself. The present study therefore achieved the first demonstration of the presence of BARD1 in body fluids, including urine. uBARD1 was upregulated in the stage I and gradually decreased depending on disease progression, suggesting that uBARD1 is suitable for early detection of GC. However, since the actual significance and mechanisms of BARD1 related to GC remain unclear, further investigation is required in the future.

In the current study, serum levels of TFF1 and BARD1 were also diagnostic biomarkers for GC. However, urinary levels of TFF1, ADAM12 and BARD1 showed better diagnostic power for GC than serum levels of them. In addition to its completely non-invasive manner, the current urinary biomarker panel thus gained an advantage over serum biomarker using these three proteins.

The most attractive point of the present biomarker is the potential for early discovery, with a good value of around AUC = 0.85. Among the 84 stage I GC patients in the present study, 83 patients were stage IA and only one patient was stage IB. Surprisingly, most stage IA patients could undergo endoscopic resection in the present study. Consequently, the present biomarker panel may enable very early detection of GC in a completely non-invasive way, contributing to not only high curability, but also high quality of life. These results from a large cohort might also confer a huge impact as a novel diagnostic biomarker. Moreover, the present biomarker panels consist of only three factors, depending on sex (uTFF1/uADAM12/*H. pylori* status for males, uTFF1/uBARD1/*H. pylori* status for females). These panels provided better sensitivity than *H. pylori* test alone. This simple combination is easily applicable to clinical use with low cost. We therefore believe that the present, non-invasive urinary biomarker panel is definitely useful in diagnostic screening for GC.

The current study has the following limitations. First, *H. pylori*-positive HCs showed a very low frequency and most negative HCs have undergone successful eradication, reflecting a recent Japanese policy to eradicate *H. pylori* for non-GC patients. Likewise, the current guidelines recommending *H. pylori* eradication will result in reduced *H. pylori* positivity in the future. However, our established urinary biomarkers were independent from *H. pylori* infection, and those urinary levels were significantly higher in GC patients than in HCs, in both *H. pylori*-positive and -negative groups. The current biomarker panel can also fit the new era of low frequency of active *H. pylori* infection. Second, we cannot completely exclude influences of other benign comorbidities. Since the present study is age- and sex-matched case-control study that showed consistent results between independent training and validation cohorts, we believe the influence from other diseases would be negligible. Third, whether these biomarkers are specific to GC is also challenging issue. However, stomach specificity of TFF1 and *H. pylori* would be able to support GC specificity of the current biomarker panel.

In conclusion, the current completely non-invasive urinary biomarker panel provides a promising method for identifying the presence of GC, as well as early detection.

## Supplementary information

Supplementary Figure and Table

## Data Availability

The data that support the findings of this study are available on request from the corresponding author (T.S.).

## References

[1] Ferlay J, Soerjomataram I, Dikshit R, Eser S, Mathers C, Rebelo M (2015). Cancer incidence and mortality worldwide: sources, methods and major patterns in GLOBOCAN 2012. Int. J. Cancer.

[2] Nashimoto A, Akazawa K, Isobe Y, Miyashiro I, Katai H, Kodera Y (2013). Gastric cancer treated in 2002 in Japan: 2009 annual report of the JGCA nationwide registry. Gastric Cancer.

[3] Uemura N, Okamoto S, Yamamoto S, Matsumura N, Yamaguchi S, Yamakido M (2001). Helicobacter pylori infection and the development of gastric cancer. N. Engl. J. Med..

[4] Sturgeon CM, Duffy MJ, Hofmann BR, Lamerz R, Fritsche HA, Gaarenstroom K (2010). National Academy of Clinical Biochemistry Laboratory Medicine Practice Guidelines for use of tumor markers in liver, bladder, cervical, and gastric cancers. Clin. Chem..

[5] Iwasaki H, Shimura T, Kataoka H (2019). Current status of urinary diagnostic biomarkers for colorectal cancer. Clin. Chim. Acta.

[6] Shimura T, Iwasaki H, Kitagawa M, Ebi M, Yamada T, Yamada T (2019). Urinary cysteine-rich protein 61 and trefoil factor 3 as diagnostic biomarkers for colorectal cancer. Transl. Oncol..

[7] Iwasaki H, Shimura T, Yamada T, Okuda Y, Natsume M, Kitagawa M (2019). A novel urinary microRNA biomarker panel for detecting gastric cancer. J. Gastroenterol..

[8] Shimura T, Ebi M, Yamada T, Yamada T, Katano T, Nojiri Y (2017). Urinary kallikrein 10 predicts the incurability of gastric cancer. Oncotarget.

[9] Shimura T, Dagher A, Sachdev M, Ebi M, Yamada T, Yamada T (2015). Urinary ADAM12 and MMP-9/NGAL complex detect the presence of gastric cancer. Cancer Prev. Res. (Philos.)..

[10] McShane LM, Altman DG, Sauerbrei W, Taube SE, Gion M, Clark GM (2005). Reporting recommendations for tumor marker prognostic studies. J. Clin. Oncol..

[11] Vandenbroucke JP, von Elm E, Altman DG, Gotzsche PC, Mulrow CD, Pocock SJ (2007). Strengthening the reporting of observational studies in epidemiology (STROBE): explanation and elaboration. Epidemiology.

[12] Sobin, L. H., Gospodarowicz, M. K. & Wittekind, C. *TNM Classification of Malignant Tumours*, 7th edn. (John Wiley & Sons, Inc., Hoboken, 2009).

[13] Wong WM, Poulsom R, Wright NA (1999). Trefoil peptides. Gut.

[14] Lefebvre O, Chenard MP, Masson R, Linares J, Dierich A, LeMeur M (1996). Gastric mucosa abnormalities and tumorigenesis in mice lacking the pS2 trefoil protein. Science.

[15] Machado JC, Carneiro F, Ribeiro P, Blin N, Sobrinho-Simoes M (1996). pS2 protein expression in gastric carcinoma. An immunohistochemical and immunoradiometric study. Eur. J. Cancer.

[16] Muller W, Borchard F (1993). pS2 protein in gastric carcinoma and normal gastric mucosa: association with clincopathological parameters and patient survival. J. Pathol..

[17] Soutto M, Belkhiri A, Piazuelo MB, Schneider BG, Peng D, Jiang A (2011). Loss of TFF1 is associated with activation of NF-kappaB-mediated inflammation and gastric neoplasia in mice and humans. J. Clin. Invest..

[18] Soutto M, Chen Z, Bhat AA, Wang L, Zhu S, Gomaa A (2019). Activation of STAT3 signaling is mediated by TFF1 silencing in gastric neoplasia. Nat. Commun..

[19] Aikou S, Ohmoto Y, Gunji T, Matsuhashi N, Ohtsu H, Miura H (2011). Tests for serum levels of trefoil factor family proteins can improve gastric cancer screening. Gastroenterology.

[20] Dardaei L, Shahsavani R, Ghavamzadeh A, Behmanesh M, Aslankoohi E, Alimoghaddam K (2011). The detection of disseminated tumor cells in bone marrow and peripheral blood of gastric cancer patients by multimarker (CEA, CK20, TFF1 and MUC2) quantitative real-time PCR. Clin. Biochem..

[21] Yamanari T, Sugiyama H, Tanaka K, Morinaga H, Kitagawa M, Onishi A (2018). Urine trefoil factors as prognostic biomarkers in chronic kidney disease. Biomed. Res. Int..

[22] Lebherz-Eichinger D, Tudor B, Ankersmit HJ, Reiter T, Haas M, Roth-Walter F (2015). Trefoil factor 1 excretion is increased in early stages of chronic kidney disease. PLoS ONE.

[23] Shimura T, Kataoka H, Ogasawara N, Kubota E, Sasaki M, Tanida S (2008). Suppression of proHB-EGF carboxy-terminal fragment nuclear translocation: a new molecular target therapy for gastric cancer. Clin. Cancer Res..

[24] Shimura T, Yoshida M, Fukuda S, Ebi M, Hirata Y, Mizoshita T (2012). Nuclear translocation of the cytoplasmic domain of HB-EGF induces gastric cancer invasion. BMC Cancer.

[25] Shakya R, Szabolcs M, McCarthy E, Ospina E, Basso K, Nandula S (2008). The basal-like mammary carcinomas induced by Brca1 or Bard1 inactivation implicate the BRCA1/BARD1 heterodimer in tumor suppression. Proc. Natl Acad. Sci. USA.

[26] Irminger-Finger I, Leung WC, Li J, Dubois-Dauphin M, Harb J, Feki A (2001). Identification of BARD1 as mediator between proapoptotic stress and p53-dependent apoptosis. Mol. Cell..

[27] Wu JY, Vlastos AT, Pelte MF, Caligo MA, Bianco A, Krause KH (2006). Aberrant expression of BARD1 in breast and ovarian cancers with poor prognosis. Int. J. Cancer.

[28] Liao Y, Yuan S, Chen X, Zhu P, Li J, Qin L (2017). Up-regulation of BRCA1-associated RING domain 1 promotes hepatocellular carcinoma progression by targeting Akt signaling. Sci. Rep..

[29] Zhang YQ, Pilyugin M, Kuester D, Leoni VP, Li L, Casula G (2012). Expression of oncogenic BARD1 isoforms affects colon cancer progression and correlates with clinical outcome. Br. J. Cancer.

[30] Pilyugin M, Descloux P, Andre PA, Laszlo V, Dome B, Hegedus B (2017). BARD1 serum autoantibodies for the detection of lung cancer. PLoS ONE.

