# Supplementary material for: Novel urinary protein biomarker panel for early diagnosis of gastric cancer
Source: Br J Cancer. 2020 Sep 16;123(11):1656–64. doi: 10.1038/s41416-020-01063-5 (PMC7686371; doi:10.1038/s41416-020-01063-5)
Supplement: Supplementary file 1 — Supplementary Figure and Table [file 41416_2020_1063_MOESM1_ESM.pdf]

Supplementary Figure

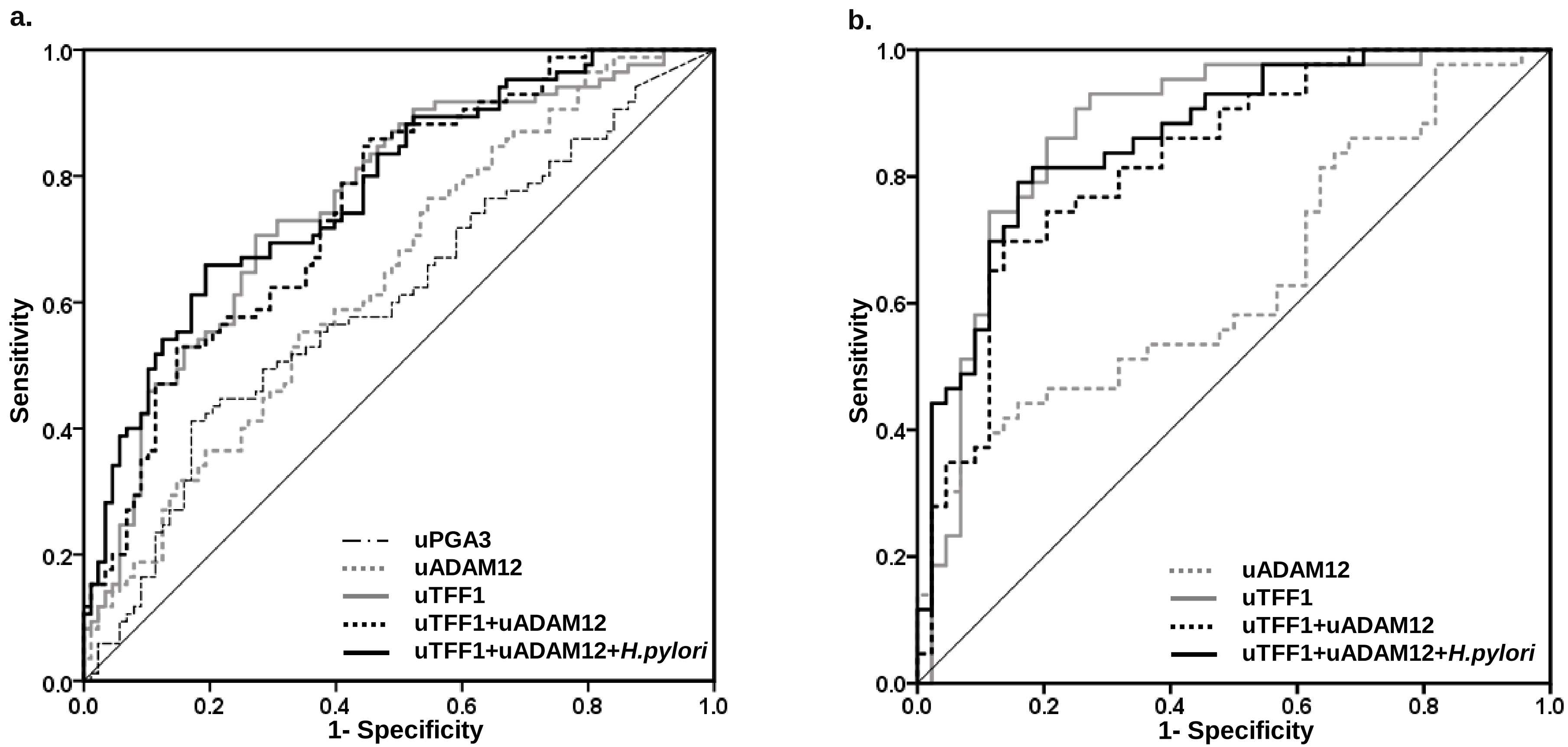

Normalized with creatinine	AUC (95%CI)	*P*
**uPGA3**	0.603 (0.518-0.688)	0.019
**uADAM12**	0.639 (0.558-0.721)	0.002
**uTFF1**	0.759 (0.687-0.830)	<0.001
**uTFF1+uADAM12**	0.752 (0.681-0.824)	<0.001
**uTFF1+uADAM12+*H. pylori***	0.777 (0.709-0.845)	<0.001

Normalized with creatinine	AUC (95%CI)	*P*
**uADAM12**	0.640 (0.523-0.757)	0.024
**uTFF1**	0.872 (0.792-0.951)	<0.001
**uTFF1+uADAM12**	0.825 (0.737-0.912)	<0.001
**uTFF1+uADAM12+*H. pylori***	0.863 (0.787-0.939)	<0.001

Fig. S1. Urinary protein biomarkers normalized to urinary creatinine
a. Training cohort; b. Validation cohort
ROC curves were obtained from values normalized to urinary creatinine.

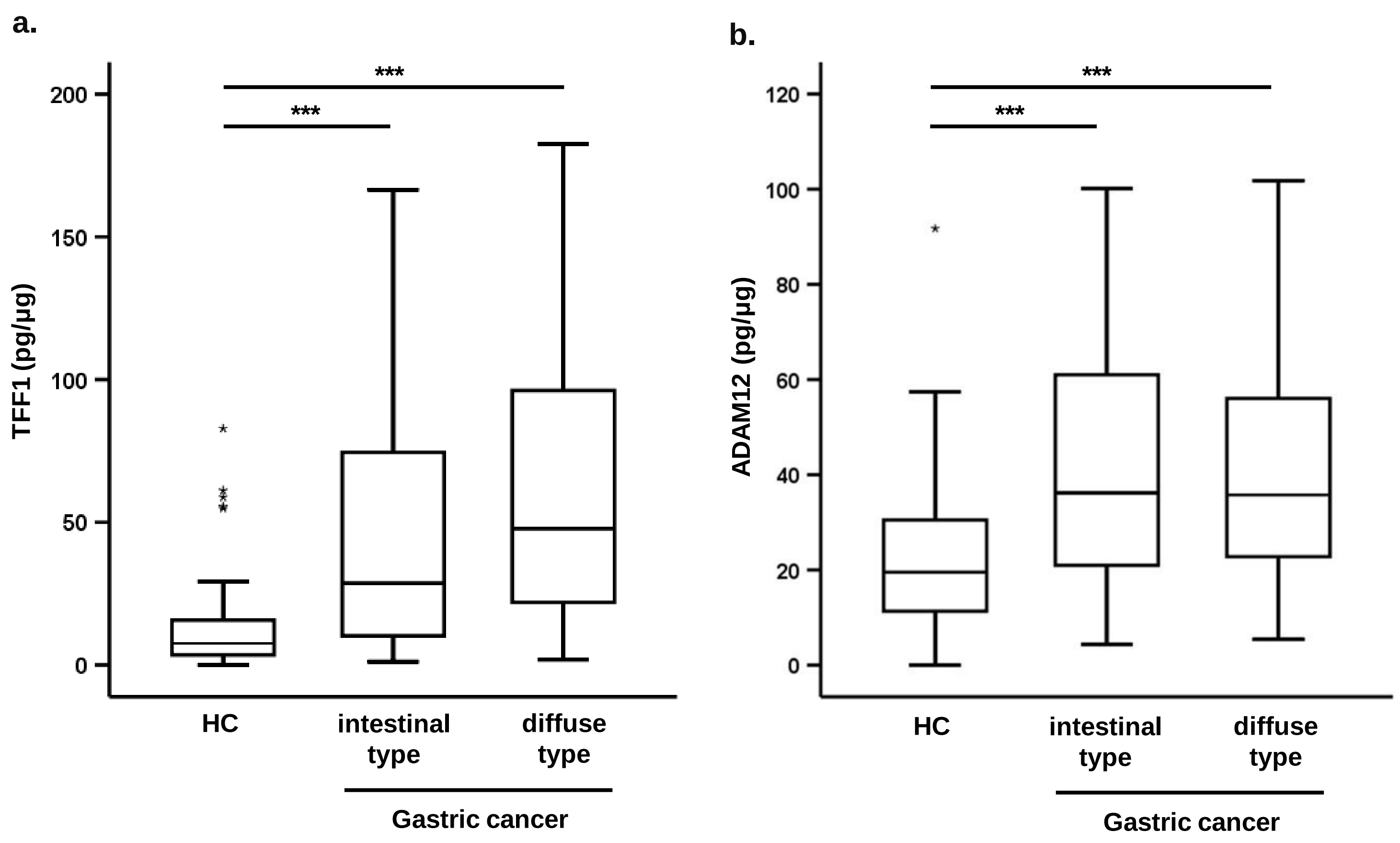

Fig. S2. Urinary levels according to Histological type
a. Urinary TFF1; b. Urinary ADAM12
The whole cohort was used for the analyses related to urinary levels of TFF1 and ADAM 12.
***, $P < 0.001$

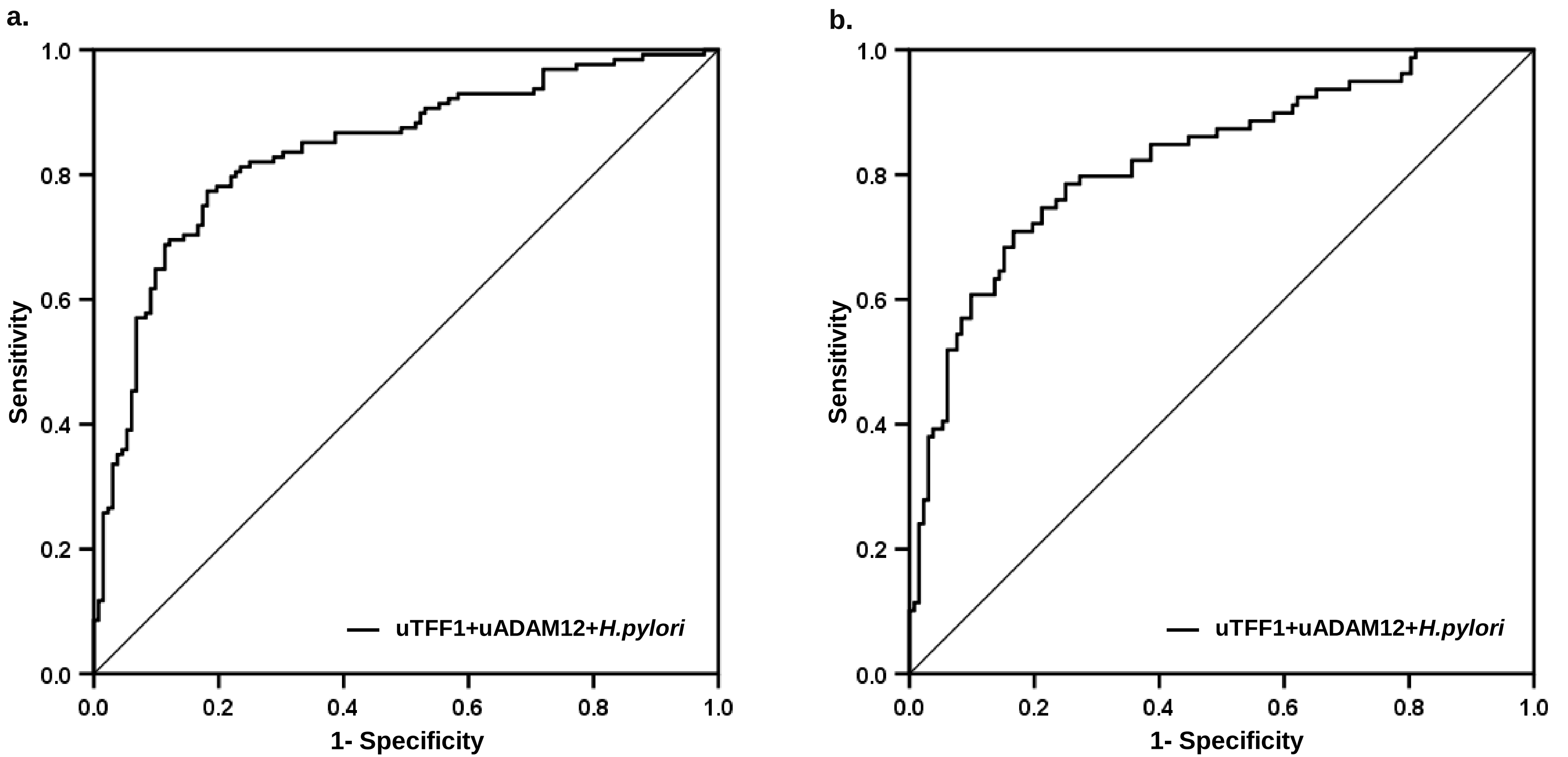

Normalized with protein	AUC (95%CI)	*P*
**uTFF1+uADAM12+*H. pylori***	0.840 (0.791-0.889)	<0.001

Normalized with protein	AUC (95%CI)	*P*
**uTFF1+uADAM12+*H. pylori***	0.824 (0.765-0.883)	<0.001

Fig. S3. Urinary protein biomarker panel according to *H. pylori* subcategorization
a. ROC curve for all stage gastric cancer; b. ROC curve for stage I gastric cancer
The *H. pylori* status was subcategorized into 3 groups: *H. pylori* positive; *H. pylori* eradicated; *H. pylori* never infected.
ROC curves were obtained from uTFF1 and uADAM12 values normalized to urinary protein and the 3 subcategorized *H. pylori* status.

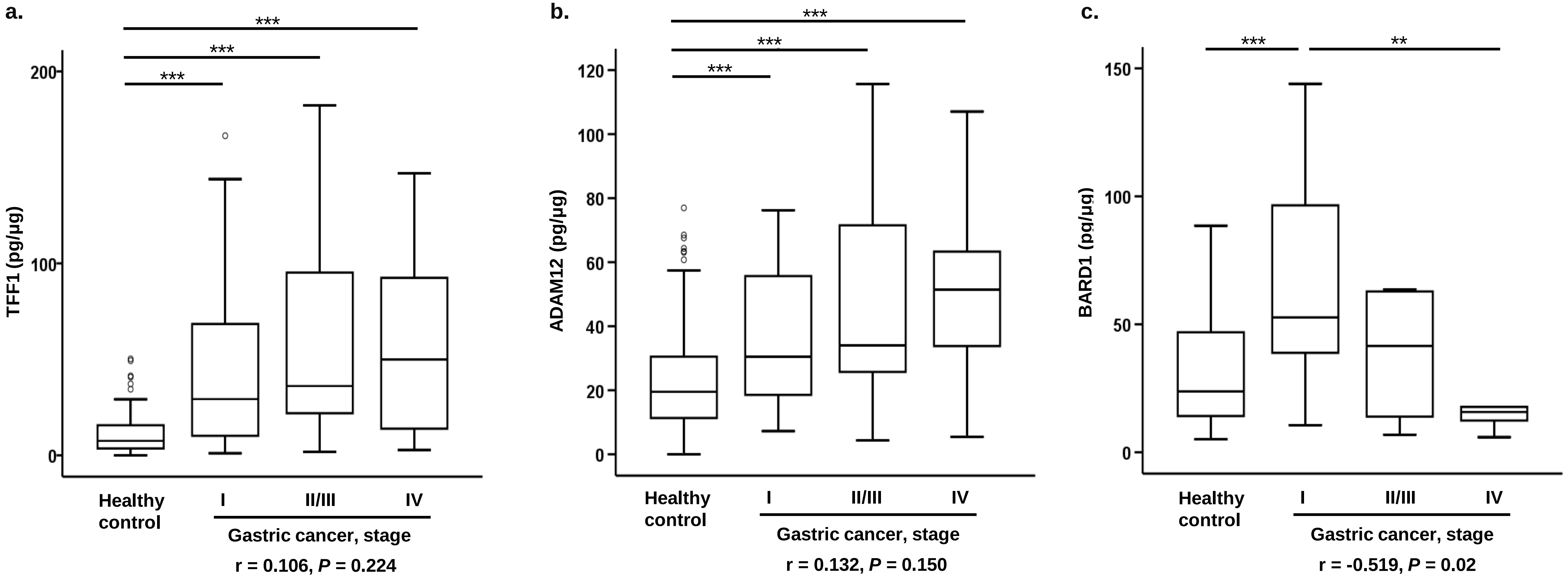

Fig. S4. Urinary levels according to disease stage
a. Urinary TFF1; b. Urinary ADAM12; c. Urinary BARD1
All gastric cancer cohorts were used for the analyses related to urinary levels of TFF1 and ADAM 12, whereas the female GC cohort was used for the analysis of urinary BARD1. Each urinary protein level was normalized to urinary total protein.
Each comparison between stages was analyzed with Mann-Whitney U test. **, *P*=0.001; ***, *P*<0.001
Correlation between each urinary level and cancer stage was r =0.106, *P*=0.224 for uTFF1, r =0.132, *P*=0.150 for uADAM12 and r=-0.519, *P*=0.02 for uBARD1, respectively. Correlation was analyzed with Spearman rank correlations.

Supplementary Table

Table S1. Urinary protein level normalized to urinary creatinine

Training cohort (n=176)						
		**Univariate analysis**			**Multivariate analysis**	
		**HC (n=88)** (Median (IQR))	**GC (n=88)** (Median (IQR))	***P***	**Odds ratio** (95%CI)	***P***
TFF1	(ng/g.Cr)	654 (386-1546)	2306 (912-4986)	<0.001	1.002 (1.000-1.003)	0.018
ADAM12	(μg/g.Cr)	2.11 (1.30-3.53)	2.90 (1.93-5.06)	0.003	1.002 (1.000-1.003)	0.028
PGA3	(ng/g.Cr)	370 (140-780)	660 (220-2520)	0.004		
BARD1	(μg/g.Cr)	3.35 (2.53-4.78)	3.49 (2.33-5.35)	0.556		
CCDC38	(μg/g.Cr)	3.96 (2.99-5.42)	3.44 (2.40-5.46)	0.200		
TINAGL1	(ng/g.Cr)	0.0 (0.0-150)	0.0 (0.0-170)	0.887		
NDRG1	(μg/g.Cr)	31 (17-53)	31 (10-68)	0.960		
DDX55	(μg/g.Cr)	6.17 (4.48-8.82)	6.11 (3.40-9.41)	0.586		
H. pylori				<0.001	4.464 (1.192-10.309)	0.001

Validation cohort (n=88)						
		**Univariate analysis**			**Multivariate analysis**	
		**HC (n=44)** (Median (IQR))	**GC (n=44)** (Median (IQR))	***P***	**Odds ratio** (95%CI)	***P***
TFF1	(ng/g.Cr)	1153 (710-1800)	4468 (2547-7650)	<0.001	1.001 (0.999-1.003)	0.207
ADAM12	(μg/g.Cr)	2.50 (1.70-3.19)	2.85 (2.08-7.68)	0.017	1.003 (1.000-1.006)	0.035
H. pylori				<0.001	9.804 (2.825-33.333)	<0.001

Table S2. Subset analysis according to *H. pylori* subcategorization

**Training cohort (n=176)**			
		**Multivariate analysis**	
		**Odds ratio** (95%CI)	***P***
TFF1	(pg/μg)	1.033 (1.010-1.057)	0.005
ADAM12	(pg/μg)	1.036 (1.017-1.056)	<0.001
H. pylori	Never infected	1	
	Eradicated	3.534 (1.294-9.709)	0.014
	Positive	4.566 (1.464-14.286)	0.009
**Validation cohort (n=88)**			
		**Multivariate analysis**	
		**Odds ratio** (95%CI)	***P***
TFF1	(pg/μg)	1.010 (0.997-1.023)	0.236
ADAM12	(pg/μg)	1.034 (1.006-1.064)	0.029
H. pylori	Never infected	1	
	Eradicated	8.772 (2.463-31.250)	0.001
	Positive	28.571 (4.524-200.0)	<0.001
**Whole cohort (n=264)**			
		**Multivariate analysis**	
		**Odds ratio** (95%CI)	***P***
TFF1	(pg/μg)	1.033 (1.010-1.057)	0.005
ADAM12	(pg/μg)	1.036 (1.017-1.056)	<0.001
H. pylori	Never infected	1	
	Eradicated	3.534 (1.294-9.709)	0.014
	Positive	4.566 (1.464-14.286	0.009

Table S3. Urinary protein biomarkers according to sex

		Male			Female		
		Univariate analysis	Multivariate analysis		Univariate analysis	Multivariate analysis	
		P	**OR** (95%CI)	***P***	***P***	**OR** (95% CI)	***P***
TFF1	(pg/μg)	<0.001	1.027 (1.005-1.051)	0.019	<0.001	1.104 (1.038-1.174)	0.002
ADAM12	(pg/μg)	<0.001	1.025 (1.007-1.043)	0.004	<0.001		
PGA3	(pg/μg)	0.109			0.003		
BARD1	(pg/μg)	0.072			0.031	1.029 (1.002-1.056)	0.033
CCDC38	(pg/μg)	0.628			0.633		
TINAGL1	(pg/μg)	0.861			0.310		
NDRG1	(pg/μg)	0.719			0.243		
DDX55	(pg/μg)	0.353			0.105		
H. pylori		<0.001	4.831 (1.675-14.085)	0.004	0.020	7.246 (1.712-30.303)	0.007

Table S4. Diagnostic power of sex-specific urinary protein biomarker panel

	Sensitivity	Specificity	Accuracy
For male GC (uTFF1+uADAM12+*H.pylori*)	88.4 % (84/95)	57.4 % (58/101)	72.4 % (142/196)
For female GC (uTFF1+uBARD1+*H.pylori*)	81.8 % (27/33)	74.1 % (23/31)	78.1 % (50/64)
**Total**	**86.7 % (111/128)**	**61.4 % (81/132)**	**73.8 % (192/260)**

Male GC predictive calculation = 5 * uTFF1 (pg/μg) + 3 * uADAM12 (pg/μg)

When *H. pylori* is negative, the cut-off value of "male GC predictive calculation" is set at ≥ 131 (pg/μg) for predicting the presence of GC.

When *H. pylori* is positive, the cut-off value of "male GC predictive calculation" is set at ≥ 136 (pg/μg) for predicting the presence of GC.

Female GC predictive calculation = uTFF1 (pg/μg) + 2.1 * uBARD1 (pg/μg)

When *H. pylori* is negative, the cut-off value of "female GC predictive calculation" is set at ≥ 112 (pg/μg) for predicting the presence of GC.

When *H. pylori* is positive, the cut-off value of "female GC predictive calculation" is set at ≥ 121 (pg/μg) for predicting the presence of GC.

Table S5. Serum levels of TFF1, ADAM12 and BARD1

**Male + Female**		**HC (n=56)** (Median (IQR))	**GC (n=37)** (Median (IQR))	***P***	**AUC (95 %CI)**	***P***
TFF1	(pg/ml)	112 (79-166)	205 (101-380)	0.004	0.676 (0.560-0.791)	0.004
ADAM12	(pg/ml)	501 (226-1345)	346 (217-720)	0.142	0.410 (0.294-0.526)	0.142
BARD1	(ng/ml)	4.57 (4.35-4.67)	4.80 (4.53-5.09)	0.001	0.703 (0.588-0.818)	0.001
**Male**		**HC (n=46)** (Median (IQR))	**GC (n=25)** (Median (IQR))	***P***	**AUC (95 %CI)**	***P***
TFF1	(pg/ml)	113 (89-171)	189 (87-353)	0.094	0.621 (0.474-0.768)	0.094
ADAM12	(pg/ml)	660 (245-1960)	434 (240-775)	0.187	0.405 (0.270-0.539)	0.187
**Female**		**HC (n=10)** (Median (IQR))	**GC (n=12)** (Median (IQR))	***P***	**AUC (95 %CI)**	***P***
TFF1	(pg/ml)	96 (71-144)	297 (122-387)	0.007	0.842 (0.679-1.000)	0.007
BARD1	(ng/ml)	3.92 (3.61-4.41)	4.52 (4.39-4.72)	0.043	0.758 (0.542-0.975)	0.041
